# Positional variability of a small intestinal stromal tumor: a case report

**DOI:** 10.1093/jscr/rjaf874

**Published:** 2025-11-06

**Authors:** Song Luo, Lieming Xv, Chuanli Huang

**Affiliations:** Department of Radiology, Jiangshan Hospital of Traditional Chinese Medicine, 38 Jiangbin Rd, Jiangshan, Quzhou, Zhejiang 324103, China; Department of Surgery, Jiangshan Hospital of Traditional Chinese Medicine, 38 Jiangbin Rd, Jiangshan, Quzhou, Zhejiang 324103, China; Department of Radiology, Jiangshan Hospital of Traditional Chinese Medicine, 38 Jiangbin Rd, Jiangshan, Quzhou, Zhejiang 324103, China

**Keywords:** gastrointestinal intestinal stromal tumor, medical imaging, tumor displacement

## Abstract

Gastrointestinal stromal tumors are rarely encountered within the pelvic cavity. Their positional variability on medical imaging poses significant diagnostic and surgical planning challenges. This report details a case in which a small intestinal stromal tumor exhibited discernible migration on serial imaging within a 24-hour period. The diagnosis was ultimately confirmed through surgical excision and subsequent histopathological examination.

## Introduction

Gastrointestinal stromal tumors (GISTs) are rare neoplasms with an estimated annual incidence of ~1 per 100 000 individuals [1]. Despite their rarity, GISTs represent the most common mesenchymal tumors of the gastrointestinal tract, with a median age at diagnosis typically ranging from 60 to 65 years. These tumors arise from mutations in the tyrosine-protein kinase (KIT) or platelet-derived growth factor receptor alpha (PDGFRA) genes [2]. Small intestinal stromal tumors (SISTs) constitute ~20%–35% of all GIST cases. Common clinical manifestations include abdominal pain, bloating, early satiety, palpable abdominal masses, gastrointestinal bleeding, and fatigue secondary to anemia. Some patients may present with acute abdominal symptoms resulting from tumor rupture or intestinal obstruction. The most frequent malignant manifestations include hepatic metastases and peritoneal dissemination, whereas lymph node involvement is remarkably uncommon. Pulmonary and extra-abdominal metastases generally occur only in advanced disease stages. The 5-year survival rate for malignant GISTs ranges from 35% to 65%, with prognosis strongly correlated to tumor size, mitotic rate, and anatomical location. Local invasion and distant metastasis serve as reliable indicators of malignant behavior. Surgical resection remains the primary treatment modality [1]. In the present case, initial computed tomography (CT) imaging suggested a pelvic mass, while subsequent magnetic resonance imaging (MRI) demonstrated tumor displacement to the lower abdominal region. These imaging findings illustrate the diagnostic challenge in determining the tumor origin, which was ultimately confirmed as a GIST through histopathological examination.

## Case report

**Figure 1:**
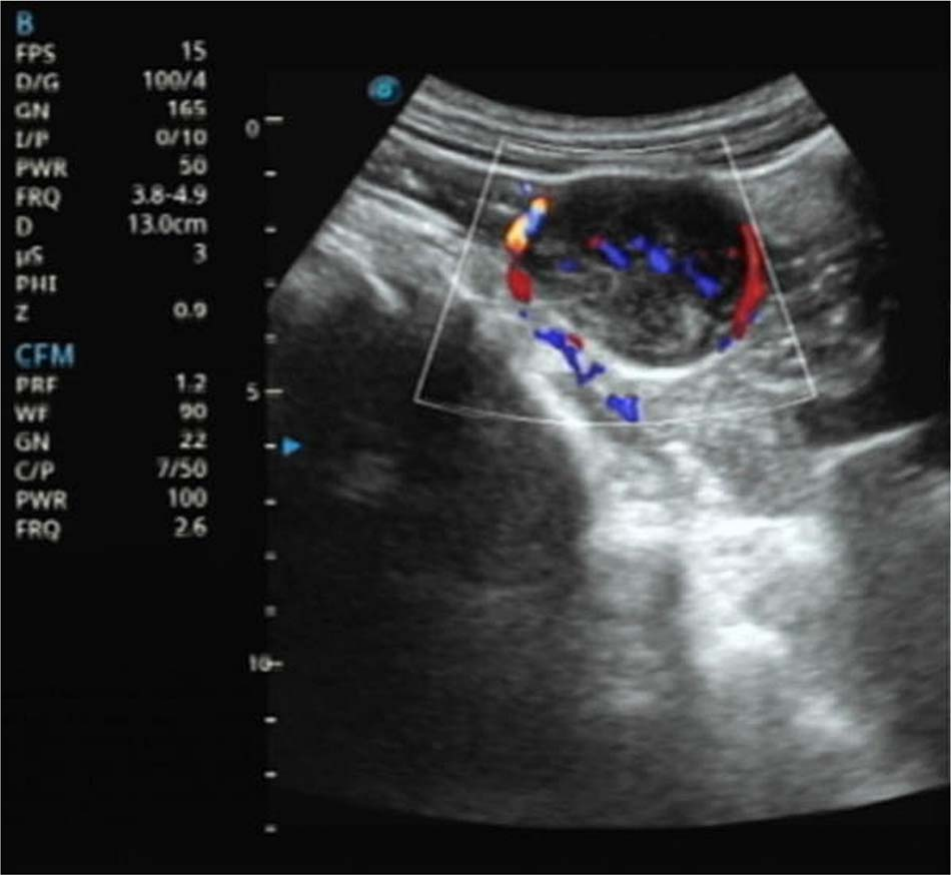
Ultrasonography demonstrates a predominantly hypoechoic mass with peripheral and intralesional blood flow signals.

A 61-year-old male presented with dysuria and was subsequently admitted for further management after an incidental 4 cm abdominal mass was detected during routine bladder ultrasonography (Fig. 1). The patient’s vital signs and abdominal examination were unrevealing. Laboratory investigations revealed a full blood count with red blood cell count (RBC) 4.72 × 10^12^/l, white blood cell count (WBC) 10.56 × 10^9^/l, and platelet count (PLT) 288 × 10^9^/l, indicating no anemia, mild leukocytosis, and a normal platelet count. Renal profile showed serum creatinine 62.9 μmol/l, blood urea nitrogen 3.73 mmol/l, sodium 140.4 mmol/l, chloride 102.2 mmol/l, and bicarbonate 32.3 mmol/l, all within acceptable ranges. Additional laboratory results included C-reactive protein (CRP) 52.46 mg/dl, total bilirubin (TBIL) 30.0 μmol/l, indirect bilirubin (IBIL) 23.4 μmol/l, carcinoembryonic antigen (CEA) 5.16 ng/ml, and carbohydrate antigen 19–9 (CA19–9) 56.26 ng/ml. Serological testing for hepatitis B revealed HBsAg >250 IU/ml (positive), HBsAb 0.317 mIU/ml (negative), HBeAg 0.000 IU/ml (negative), HBeAb >12.000 IU/ml (positive), and HBcAb >40.000 IU/ml (positive), consistent with chronic hepatitis B infection. Tests for hepatitis C antibody and hepatitis A IgM/IgG were negative. No other significant comorbidities or risk factors were identified.

**Figure 2:**
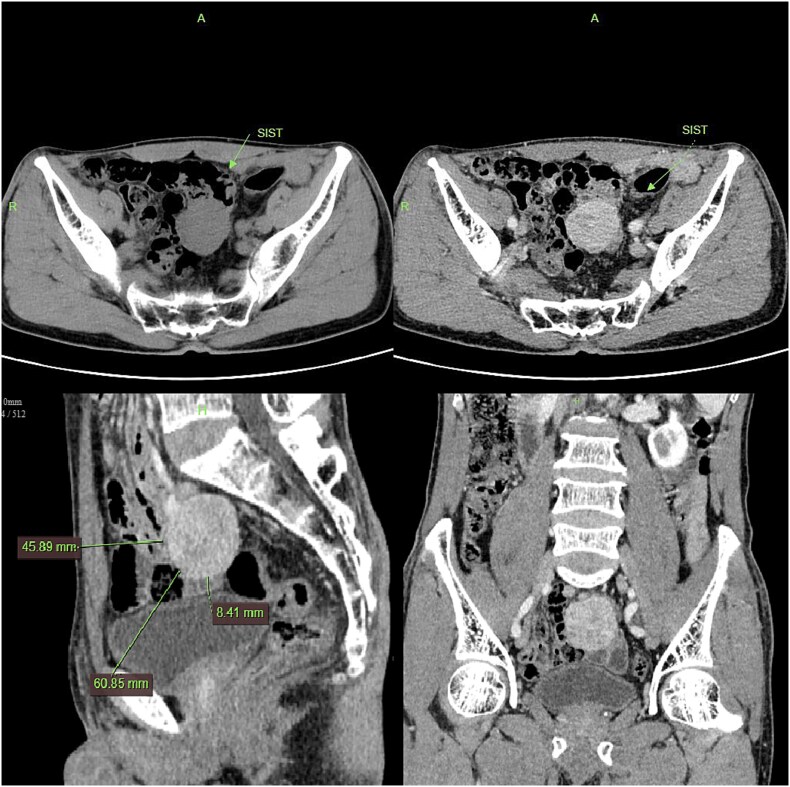
CT imaging shows a pelvic lesion with soft tissue density on non-enhanced scans, marked enhancement of the primary tumor mass after contrast, and coronal/sagittal reconstructions delineating its anatomical location.

**Figure 3:**
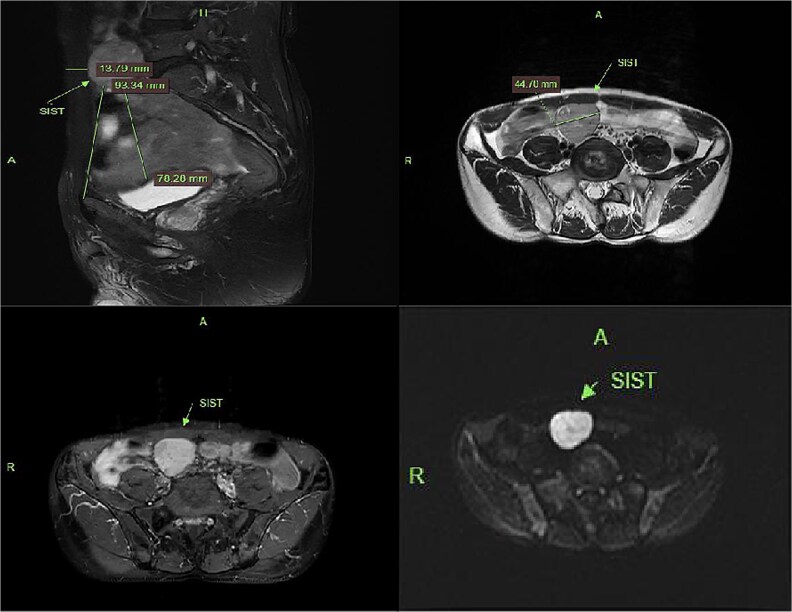
MRI demonstrates superior displacement of the tumor compared to CT, with isointense signal on T2-weighted fat-suppressed sequences, post-contrast enhancement, and hyperintensity on DWI at a b-value of 800 s/mm².

Contrast-enhanced CT demonstrated a well-circumscribed, round enhancing mass measuring 4 cm in diameter. Sagittal imaging showed the lesion positioned 0.9 cm from the bladder wall, 4.5 cm from the anterior abdominal wall, and 6.1 cm from the pubic symphysis (Fig. 2). The mass exhibited an intact capsule without evidence of rupture. MRI performed within a 24-hour period, sagittal imaging showed the lesion positioned 7.9 cm from the bladder wall, 1.3 cm from the anterior abdominal wall, and 9.3 cm from the pubic symphysis, and confirmed stable positioning and morphology, maintaining identical spatial relationships to adjacent structures (Fig. 3).

**Figure 4:**
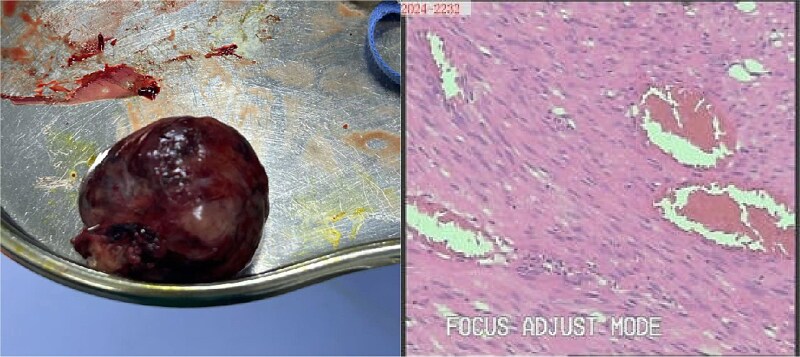
Gross specimen photograph of the laparoscopically resected tumor (right panel) with corresponding histopathological sections (left panel).

The patient underwent laparoscopic resection under general anesthesia in the lithotomy position. Intraoperative exploration identified a 4 cm ileal mass with significant omental adhesions, which were dissected using an ultrasonic scalpel. En bloc resection of the mass with adjacent ileal segment was performed. Histopathological examination confirmed a poorly differentiated GIST (Fig. 4).

## Discussion

SISTs typically follow an insidious and asymptomatic course, frequently discovered incidentally during imaging performed for unrelated indications. Preoperative diagnosis relies primarily on imaging, with relatively high diagnostic yield for larger tumors. In this case, the tumor was an incidental finding during a bladder-oriented examination. It did not cause significant bladder compression or associated symptoms, such as urinary frequency. CT provided detailed multiplanar information, revealing a pelvic mass surrounded by abundant bowel loops. Following contrast CT, the tumor exhibited significant and relatively homogeneous enhancement without calcification; these features are typical for GISTs [3]. The absence of bowel preparation, combined with intraluminal gas and indistinct perilesional omental fat, complicates differentiation from entities such as small intestinal lymphoma. MRI demonstrated anterior and superior displacement of the mass relative to its position on CT. On T1-weighted images, the lesion demonstrated low to intermediate signal intensity (SI), while T2-weighted images revealed heterogeneous SI. Fat-saturated sequences showed no evidence of low signal intensity. Diffusion-weighted imaging (DWI) and apparent diffusion coefficient (ADC) mapping confirmed the solid nature and high cellularity of the mass, characteristic features of abdominal mesenchymal tumors. Further evaluation indicated the tumor arose from the muscularis propria of an adjacent bowel loop, with a tendency to displace rather than invade surrounding structures.

Histopathological analysis remains the diagnostic gold standard for GIST. In this case, microscopic examination revealed spindle cell morphology. Immunohistochemical staining was positive for CD117 and DOG1, with a Ki-67 proliferation index of 3%. Findings were negative for SMA, Desmin, and S-100, confirming the diagnosis of GIST [4].

Surgical excision represents the most effective treatment for SISTs. Complete resection without tumor rupture and with negative histological margins is crucial for favorable patient prognosis [5]. In this instance, the displacive growth pattern and positional variability of the tumor presented substantial challenges for both radiological diagnosis and surgical planning.
